# TRIM25 Identification in the Chinese Goose: Gene Structure, Tissue Expression Profiles, and Antiviral Immune Responses In Vivo and In Vitro

**DOI:** 10.1155/2016/1403984

**Published:** 2016-11-22

**Authors:** Yunan Wei, Hao Zhou, Anqi Wang, Lipei Sun, Mingshu Wang, Renyong Jia, Dekang Zhu, Mafeng Liu, Qiao Yang, Ying Wu, Kunfeng Sun, Xiaoyue Chen, Anchun Cheng, Shun Chen

**Affiliations:** ^1^Institute of Preventive Veterinary Medicine, Sichuan Agricultural University, Chengdu, Sichuan 611130, China; ^2^Avian Disease Research Center, College of Veterinary Medicine of Sichuan Agricultural University, Chengdu, Sichuan 611130, China; ^3^Key Laboratory of Animal Disease and Human Health of Sichuan Province, Sichuan Agricultural University, Chengdu, Sichuan 611130, China

## Abstract

The retinoic acid-inducible gene I (RIG-I) and the RIG-I-like receptor (RLR) protein play a critical role in the interferon (IFN) response during RNA virus infection. The tripartite motif containing 25 proteins (TRIM25) was reported to modify caspase activation and RIG-I recruitment domains (CARDs) via ubiquitin. These modifications allow TRIM25 to interact with mitochondrial antiviral signaling molecules (MAVs) and form CARD-CARD tetramers. Goose TRIM25 was cloned from gosling lungs, which possess a 1662 bp open reading flame (ORF). This ORF encodes a predicted 554 amino acid protein consisting of a B-box domain, a coiled-coil domain, and a PRY/SPRY domain. The protein sequence has 89.25% sequence identity with* Anas platyrhynchos* TRIM25, 78.57% with* Gallus gallus* TRIM25, and 46.92% with* Homo sapiens* TRIM25. TRIM25 is expressed in all gosling and adult goose tissues examined. QRT-PCR revealed that goose TRIM25 transcription could be induced by goose IFN-*α*, goose IFN-*γ*, and goose IFN-*λ*, as well as a35 s polyinosinic-polycytidylic acid (poly(I:C)), oligodeoxynucleotides 2006 (ODN 2006), and resiquimod (R848) in vitro; however, it is inhibited in H9N2 infected goslings for unknown reasons. These data suggest that goose TRIM25 might play a positive role in the regulation of the antiviral immune response.

## 1. Introduction

The tripartite motif family (TRIM) is a large family of proteins that share a common N-terminal RBCC motif (including a RING finger, one or two B-box, and a coiled-coil domain) and are involved in many biological processes, such as cell differentiation, apoptosis, transcription regulation, and cell signaling [1]. TRIM proteins play a critical role in a number of innate immunity responses and are critical for antiviral responses [2, 3].

The innate immune system is the first line of defense against detrimental pathogens, such as bacteria and viruses. Critical innate immune responses against viruses include constitutively expressed proteins with intrinsic antimicrobial properties and the inducible type I interferon (I-IFN) system. In general, pattern recognition receptors (PRRs), such as Toll-like receptors (TLRs), retinoic acid-inducible gene I- (RIG-I-) like receptors (RLRs), and NOD-like receptors (NLRs), take the lead in recognizing the pathogen-associated molecular pattern (PAMP) when pathogens invade organisms [4, 5]. Inducible innate immune responses are subsequently activated to increase I-IFN production by triggering downstream signaling pathways with a series of adaptor proteins that transmit downstream signals. The RLR protein plays a critical role in interferon responses during RNA virus infection. Over the past years, more and more TRIM proteins have been found to initiate the innate immune response via their capacity to act as an E3-ligase. In fact, there are seven lysine residues (K6, K11, K27, K29, K33, K48, and K63) present in ubiquitin, on which polyubiquitin chains could form. Different polyubiquitin chains linked through different lysine have diverse functions [6]. K48 linked polyubiquitin chains are a signal for degradation, while the K63 linked polyubiquitin can used to activate antiviral signaling pathways [7, 8]. Upon recognizing PAMP RNA, RIG-I hydrolyzes adenosine triphosphate (ATP) and undergoes a conformational change in which the RNA binding domain unfolds for subsequent interaction with PAMP RNA, while further releasing the CARD domain for MAVs interaction triggering the IFN signaling pathway. Meanwhile, E3-ligase TRIM25 modifies the CARD domain of RIG-I via ubiquitin, which allows it to interact with MAVs to form the CARD-CARD tetramer [9, 10]. In addition, a linear ubiquitin assembly complex was found to suppress RIG-I-mediated antiviral activity by binding human TRIM25 and eventually induces its degradation [11]. Recently, a number of studies revealed that TRIM family proteins play several diverse roles in antiviral innate immune responses. The TRIM proteins could inhibit virus replication at different stages, such as viral entry, transcription of viral genes, and viral release from the cells [1, 4, 11–14]. Another study reported that TRIMs adapt to regulate the innate immune response by attaching the K(lys)-27-linked polyubiquitin chains to the NF-*κ*B essential modulator (NEMO), which in turn activates downstream signaling for the antiviral response. Kei-ichiro Arimoto recently demonstrated that TRIM23 directly interacts with NEMO and conjugates ubiquitin. Arimoto also speculated that TRIM25 sits between the tumor necrosis factor receptor associated factor 3 (TRAF3) and NEMO; however, the mechanisms underlying the transmission of signals from TRAF3 to NEMO remain unknown [15]. Despite TRIM's indirect antiviral function through innate immune response enhancement, studies have revealed that TRIMs could inhibit HIV via an ambiguous mechanism. These findings emphasize the importance of TRIM in the innate immune response above all other antiviral proteins [1, 16].

Human TRIM25 was first identified as an estrogen-responsive finger protein induced by I-IFNs in treated macrophages and dendritic cells [17]. Similarly, it was reported that the TRIM25 could be induced by I-IFN in felines [18]. Over the years, the TRIM25 gene has been identified and characterized in humans, mice, chickens, and ducks. Studies suggest that TRIM25 might be an important component of antiviral activity in chickens [19]. In ducks, it has been shown that TRIM25 is induced by the production of I-IFNs through the RIG-I pathway [20]. Recently, studies indicated that birds have a relatively small number of immune-related genes compared to mammals. Some considerably important antiviral genes, such as TLR8, interferon regulatory factor (IRF) 3, and interferon stimulated gene 15 (ISG 15), are missing in birds, while RIG-I and other RLR modifiers, such as Riplet, are missing in chickens [21]. In addition, it was reported that feline TRIM25 lacks the RING finger domain observed in the corresponding WT-TRIM25 protein resulting in a reduction in viral release, while previous studies revealed that the RING finger domain of TRIM25 plays a role in the antiviral response by mediating lysine 63-linked ubiquitination of the N-terminal CARD domains of RIG-I to promote I-IFN production [22, 23]. Therefore, it is assumed that antiviral genes, such as TRIM25, are more important in birds than in mammals. Goose TRIM25 lacks the RING finger domain. It remains unknown whether TRIM25 works as an antiviral protein in goose.

Birds have a long evolutionary history. With the discovery of the* Archaeopteryx*, more and more people consider birds the direct descendants of dinosaurs. The sequencing of goose TRIM25 provides a better understanding of the avian immune system. In this study, we cloned full length cDNA of the goose TRIM25 gene and investigated its tissue distribution in both goslings and adult geese. Furthermore, we analyzed the transcription levels of TRIM25 in geese infected with the avian influenza A (H9N2) virus and in peripheral blood mononuclear cells (PBMCs) treated with H9N2 and gosling plague virus (GPV). TRIM25 transcription levels were also assessed in goose embryo fibroblasts (GEFs) stimulated with poly(I:C), ODN2006, R848, and goose IFN protein. Our study provides essential information on goose TRIM25 that can inform future studies investigating the interplay between bird TRIM25 and RIG-I. This study also elucidates the mechanisms by which TRIM25 activates the RIG-I signaling pathway to induce the production of interferon.

## 2. Materials and Methods

### 2.1. Virus and Animal Ethics Statement

All geese and goose embryos used in this study were purchased from the Sichuan Agricultural University Farm. The H9N2 (7.14 × 10^12.64^ copies/mL) was kindly provided by the Shanghai Veterinary Research Institute, Chinese Academy of Agricultural Sciences. The GPV (10^−6.6^ EID_50_/0.2 mL) strain was provided by the Avian Disease Research Center of Sichuan Agriculture University. The animal studies were approved by the Institutional Animal Care and Use Committee of the Sichuan Agricultural University and followed the National Institutes of Health Guide for the Care and Use of Laboratory Animals.

### 2.2. RNA Isolation and cDNA Synthesis

First, total RNA was isolated from tissues and cells using TRIZOL (Invitrogen, USA) following the manufacturer's guidelines. Subsequently, the cDNA used for the amplification of goose TRIM25 and qRT-PCR was synthesized using the 5-All-in-one Kit (Abm, Canada) according to the manufacturer's guidelines. All steps described above were performed under RNase-free conditions.

### 2.3. Cloning of Full Length Goose TRIM25

First, the goose TRIM25 sequence was amplified using the gene-specific primers G-TRIM25-F and G-TRIM25-R, which were designed based on the predicted goose sequences obtained from GenBank. Then, the full length TRIM25 cDNA, including the short 5′-untranslated regions (UTRs), was obtained by PCR using the specific primers designed in the previous step. The amplified PCR fragments were purified using the universal DNA Purification Kit (Tiangen, China) and subcloned into pMD19-T (Sigma–Aldrich, USA), which was then transformed into high-efficiency DH5*α* competent cells. The positive clones were selected and then sent for sequencing. The full length cDNA sequence was assembled. Its continuity was confirmed by sequencing the cloned PCR product amplified with a pair of terminal primers. These primers were designed for quantitative reverse transcription polymerase chain reaction (qRT-PCR) based on the acquired sequences.  Table 1 lists all primers used in the procedures described above.

### 2.4. Cell Culture and Treatment

The GEFs were isolated from 9-day-old goose embryos and seeded into individual wells of a cell culture plate. The PBMCs were isolated from goose peripheral blood using the goose peripheral blood mononuclear cell separation medium kit (TBD, China) according to the manufacturer's guidelines. All isolated cells were cultivated in Dulbecco's modified Eagle's medium (DMEM) supplemented with 10% fetal calf serum at 37°C in a humidified 5% CO_2_ atmosphere for 24 hours. The media were supplemented with penicillin (100 U/mL) and streptomycin (100 *μ*g/mL). The PBMCs were isolated from several healthy adult geese. The PBMCs obtained from the blood of each goose were seeded into several wells of a cell culture plate. Each well contained 200 *μ*L of cell suspension with a density of 5 × 10^5^ cells/mL. Then, the GEFs were divided into 4 groups and stimulated with either poly(I:C) (Sigma–Aldrich, USA) (100 *μ*g/well), goose IFN*α* (60 *μ*L/well), goose IFN*γ* (60 *μ*L/well), or goose IFN*λ* (60 *μ*L/well) and ploy(I:C) (100 *μ*g/well). PBS (60 *μ*L/well) was chosen as the control. The PBMCs were divided into 7 groups with stimulation of H9N2 (10^3^ TCID_50_/50 *μ*L), GPV (10^4^ TCID_50_/50 *μ*L), poly(I:C) (100 *μ*g/well), IFN*α* (60 *μ*L/well), ODN2006 (100 *μ*g/well), R848 (100 *μ*g/well), or PBS (60 *μ*L/mL) as the control. Stimulation with poly(I:C), ODN2006, and R848 lasted 6 hours before the cells were collected into RNase-free centrifuge tubes with 900 *μ*L of TRIZOL, while the cells treated with goose IFN*α*, IFN*γ,* and IFN*λ* were collected 24 hours after stimulation. The recombinant goose IFNs used in this study were expressed by pcDNA3.1(+)-goIFN*α*, pcDNA3.1(+)-goIFN*γ*, and pcDNA3.1(+)-goIFN*λ* in baby hamster kidney 21 cells (BHK 21).

### 2.5. Animal Experiment

The 1-day-old goslings were fed in the animal rooms for 3 days before challenge. Then, 18 healthy goslings were randomly chosen and divided into 2 groups. The first group was inoculated with 500 *μ*L of H9N2 via intramuscular injection. The control group was inoculated with the same volume of PBS. On day 1, day 3, and day 7 after infection, three goslings from each group were randomly selected for tissue sampling (lung, small intestine, blood, bursa of Fabricius, trachea, and brain). All tissues were ground in liquid nitrogen and stored in TRIZOL. The total RNA was extracted from each tissue using the TRIZOL reagent according to the manufacturer's instructions.

In addition, the tissue distribution profile of TRIM25 was performed in both healthy adult geese and goslings. The cDNA was reverse transcribed using the 5-All-in-one Kit (Abm, Canada) according to the manufacturer's guidelines. All of the synthesized cDNA were stored at −80°C for later use.

### 2.6. The Detection of Goose TRIM25 mRNA Transcription Levels

First, the cDNA used to clone the full length TRIM25 and the mRNA used to assess the transcription levels were synthesized using the same procedures described above. Then, the qRT-PCR analysis was conducted to determine the TRIM25 transcription levels in the different organs of the experimental geese and the treated GEFs and PBMCs. Two gene-specific primers (sense primer G-TRIM25-RT-F and reverse primer G-TRIM25-RT-R (Table 1)) that amplified a 127 bp TRIM25 fragment were designed to detect the transcription levels of TRIM25. Two other primers amplifying a 172 bp *β*-actin fragment served as an internal control. The qRT-PCR reaction mixture contained 5 *μ*L of EvaGreen qPCR MasterMix (Abm, Canada), 0.3 *μ*L of each primer (10 pmol/*μ*L), 0.4 *μ*L of cDNA, and 4 *μ*L of sterile water. The qRT-PCR was performed using the Bio-Rad CFX96 Real-Time Detection System (Bio-Rad, USA). The goose TRIM25 qRT-PCR procedure was as follows: 95°C for 3 min, followed by 39 cycles of 95°C for 10 s, 57°C for 30 s (61°C for *β*-actin). Finally, the qRT-PCR data were analyzed using the 2^−ΔΔCT^ method with Bio-Rad CFX Manager Software.

### 2.7. Bioinformatic Analysis

The goose TRIM25 sequence was compared with the predicted TRIM25 sequence obtained from GenBank using the BLASTX and BLASTP search programs (http://blast.ncbi.nlm.nih.gov/Blast.cgi). The ORF and the protein domain were determined using the ORF Finder (http://www.ncbi.nlm.nih.gov/gorf/orfig.cgi/). The protein structure motif was predicted using SMART (simple modular architecture research tool, http://smart.embl-heidelberg.de/). The phylogenetic tree was created using the neighbor-joining (NJ) method in the MEGA version 5.0 software package. Bootstrap values were calculated with 1000 iterations to estimate the robustness of the internal branches.

## 3. Results

### 3.1. Clone and Sequence Analysis of Goose TRIM25

**Figure 1:**
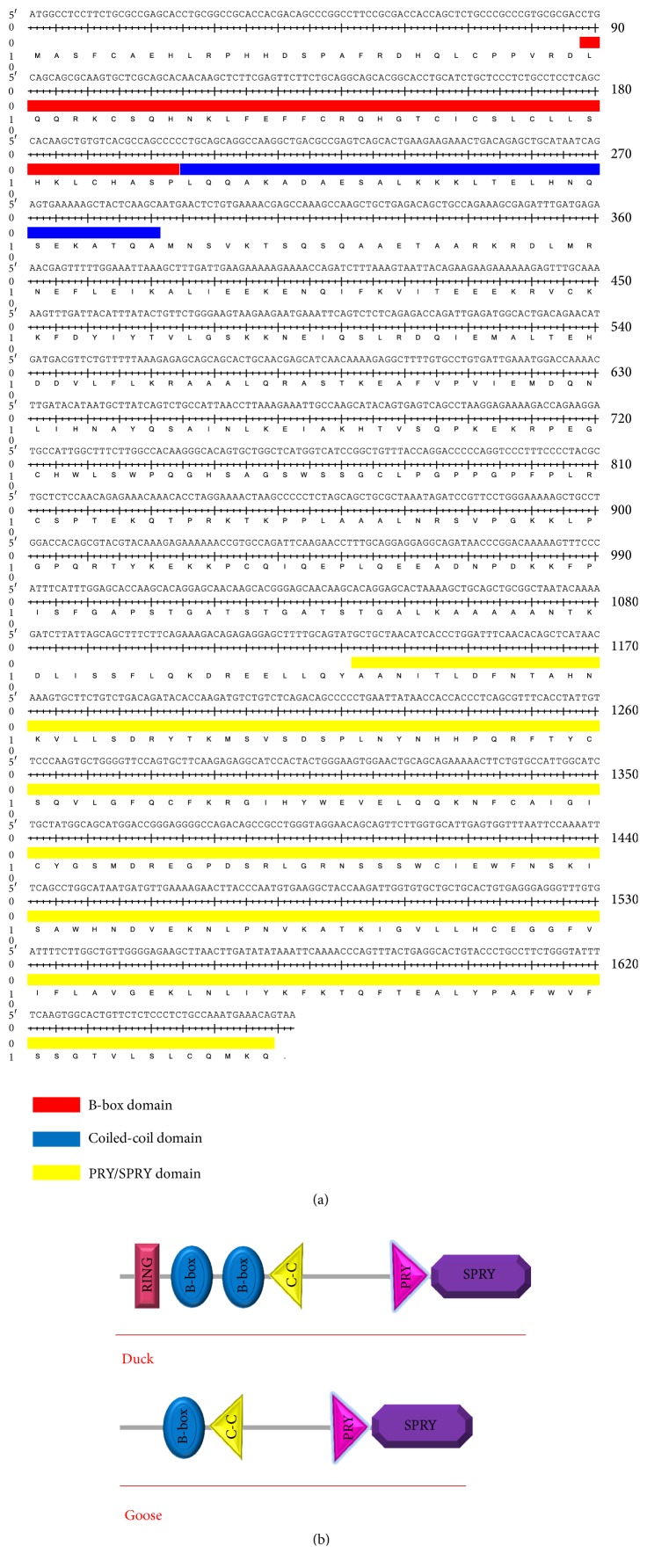
The nucleic acid and amino acid sequence of goose TRIM25 and its predicted domain. (a) The nucleic acid and predicted amino acid sequence of goose TRIM25. (b) The predicted domain of goose and* Anas platyrhynchos* TRIM25.

**Figure 2:**
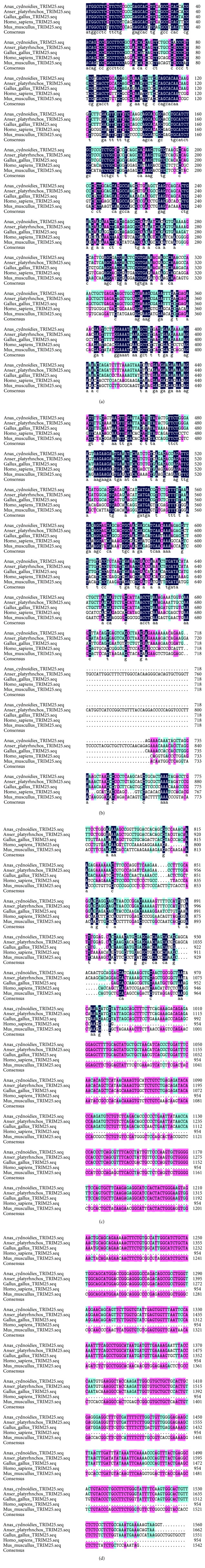
Multiple comparisons of TRIM25 from several species. Multiple comparisons of goose (*Anser cygnoides*),* Gallus gallus*,* Anas platyrhynchos*,* Homo sapiens,* and* Mus musculus* TRIM25 nucleic acid sequences. Highlighted regions indicate the homology of TRIM25 between species.

**Figure 3:**
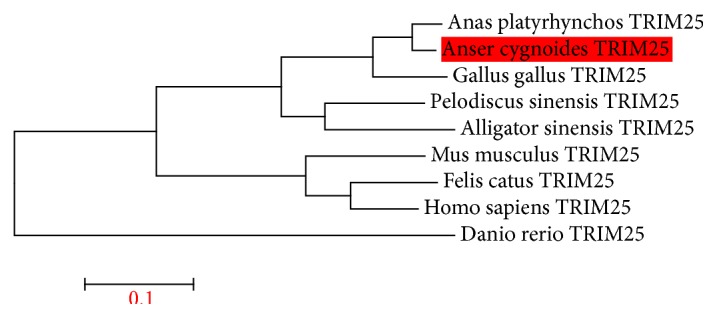
Phylogenetic tree of goose (*Anser cygnoides*) TRIM25. The tree was constructed using MEGA 6.0 with the following TRIM protein sequences:* Homo sapiens* (NM_005082.4),* Mus musculus* (NM_009546.2),* Felis catus* (NM_001290251.1),* Gallus gallus* (NM_001318548.1),* Anas platyrhynchos* (XM_013092756.1),* Danio rerio* (NM_200175.1),* Pelodiscus sinensis* (XM_00125153.2),* Alligator* (XM_006017407.2), and the* Anser cygnoides* TRIM25 cloned.

The 1892 bp cDNA of TRIM25 (KX_364385) with a 1662 bp conserved ORF was cloned (Figure 1(a)). This clone coded a predicted 554 amino acid protein. Sequence analysis by SMART program revealed that goose TRIM25 ORF contains an N-terminal BCC motif which consist of a B-box domain and a coiled-coil domain; however for unknown reason no RING finger domain exists in goose TRIM25 (Figure 1(b)). The protein sequence of goose TRIM25 had 89.25% identity with* Anas platyrhynchos* TRIM25, 78.57% with* Gallus gallus* TRIM25, and 46.92% with* Homo sapiens* TRIM25 (Figure 2). The C-terminus of goose TRIM25 contains a PRY domain followed by a SPRY domain. In addition, the phylogenetic tree analysis suggested that goose TRIM25 is closer to* Anas platyrhynchos* TRIM25 and* Gallus gallus* TRIM25 (Figure 3).

### 3.2. The Distribution of TRIM25 in Gosling and Adult Goose Tissues

**Figure 4:**
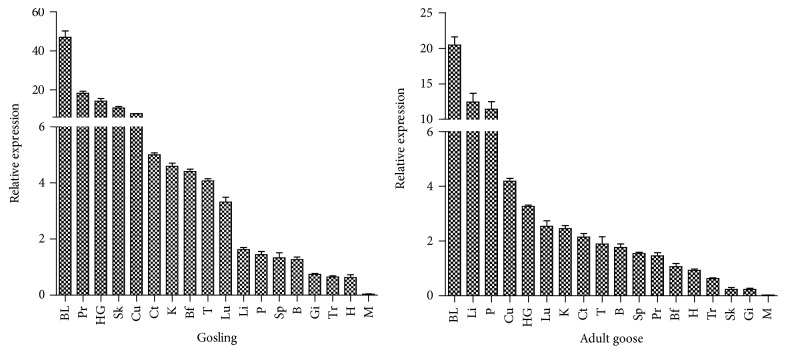
Tissue distribution profiles of goose TRIM25 in goslings and adult geese. *β*-Actin was used as the internal control gene. The expression data are represented as the mean ± SEM (*n* = 3). BL (blood), Pr (proventriculus), HG (harderian gland), SI (small intestine), Cu (caecum), Ct (caecum tonsil), K (kidney), Bf (bursa of Fabricius), T (thymus), Lu (lung), Li (liver), Sp (spleen), P (pancreas), B (brain), Gi (gizzard), Tr (trachea), H (heart), and M (muscle).

The mRNA transcription levels of gosling and adult goose TRIM25 in 18 tissues were selected for quantitative qRT-PCR. As shown in Figure 4 TRIM25 was expressed in all tissues examined in goslings and adult geese. In addition, TRIM25 was highly expressed in the blood, liver, pancreas, cecum, and harderian gland of adult geese. In goslings, TRIM25 was highly expressed in the proventriculus, harderian gland, and kidneys. However, TRIM25 expression was consistently low in the muscles of all geese examined in this study.

### 3.3. The Effects of Agonist and Goose IFNs on Goose TRIM25 Transcription Levels in Treated GEFs

**Figure 5:**
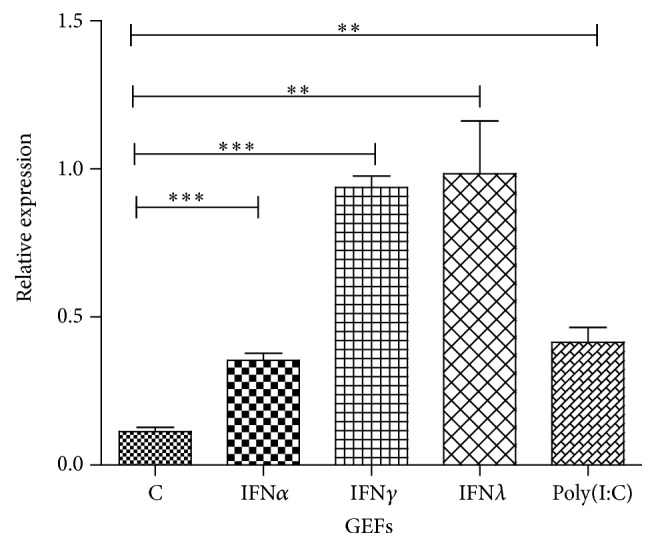
Effects of goose IFN*α*, IFN*γ*, IFN*λ*, and poly(I:C) on goose TRIM25 in GEFs. The GEFs were treated with goose IFN*α*, IFN*γ*, and IFN*λ* for 24 hours and poly(I:C) for 6 hours. The expression data are represented as the mean ± SEM (*n* = 4). Differences in mRNA cytokine production in challenged cells were analyzed using the unpaired, two-tailed *t*-test (^*∗∗*^ *P* < 0.01; ^*∗∗∗*^ *P* < 0.001).

TRIM25 is one of the interferon stimulated genes. To determine whether TRIM25 can be induced by IFNs in GEFs, the transcription levels of TRIM25 following treatment with poly(I:C) and goose IFN were determined using qRT-PCR. After 24 h of treatment with IFN (shown in Figure 5), we found that goose IFN*α* (*P* < 0.001), IFN*γ* (*P* < 0.001), and IFN*λ* (*P* < 0.01) significantly upregulated goose TRIM25 mRNA transcription levels. TRIM25 mRNA transcription levels were also upregulated in GEFs treated with poly(I:C) (*P* < 0.01).

### 3.4. The Effects of Agonist, Goose IFN*α*, and Viruses on Goose TRIM25 Transcription Levels in Treated PBMCs

**Figure 6:**
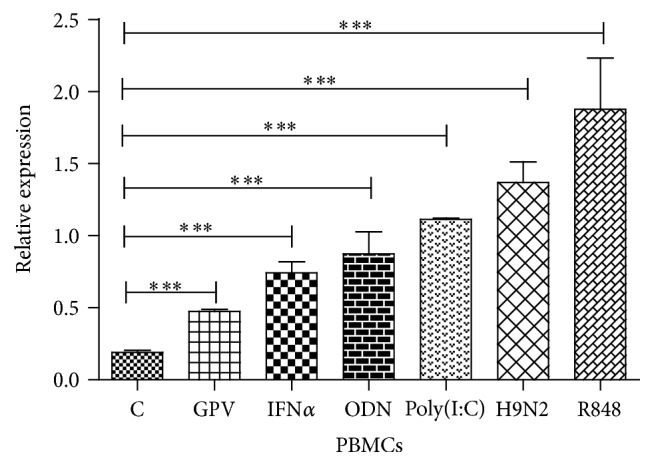
Effects of H9N2, GPV, poly(I:C), R848, ODN2006, and goose IFN*α* on goose TRIMM25 transcript levels in PBMCs. *β*-Actin was used as the internal control gene to detect the transcription levels of goose TRIM25. PBMCs were infected with either H9N2 or GPV for 24 hours. PBMCs were treated with poly(I:C), ODN2006, R848 for 6 hours, and IFN*α* for 24 hours. The expression data represent the mean ± SEM (*n* = 3). The differences in mRNA cytokine production in virus-challenged goslings and cells were analyzed using the unpaired, two-tailed *t*-test (^*∗∗∗*^ *P* < 0.001).

It was reported that viral RNA and DNA could immediately trigger antiviral responses in human and murine cells. To investigate if goose TRIM25 is involved in the antiviral response, we determined the mRNA transcription levels of goose TRIM25 in PBMCs following treatment with H9N2, GPV, poly(I:C), ODN2006, IFN*α*, and R848. We found that H9N2 (*P* < 0.01) and GPV (*P* < 0.001) infection significantly upregulated TRIM25 mRNA levels in the treated PBMCs (Figure 6). TRIM25 mRNA levels were also upregulated by IFN*α* and poly(I:C), ODN2006, and R848 treatment, which served as positive controls.

### 3.5. The Effects of H9N2 on Goose TRIM25 Transcription Levels In Vivo

**Figure 7:**
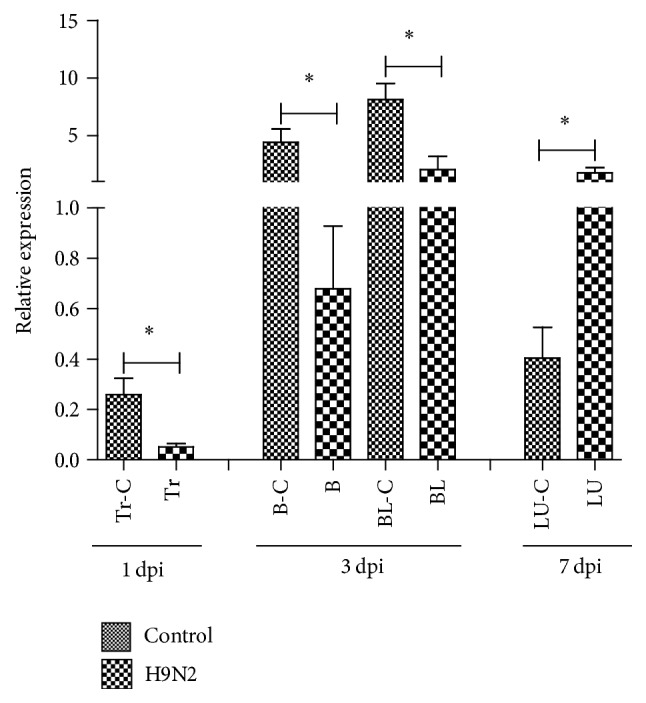
The effects of H9N2 on the expression of goose TRIM25. Expression levels of gosling TRIM25 in the blood, lungs, brain, small intestines, bursa of Fabricius, and trachea at 24 hours, 48 hours, and 72 hours after infection with H9N2 via intramuscular injection. The expression data represent the mean ± SEM (*n* = 3). Differences in mRNA cytokine production in virus-challenged goslings were analyzed using the unpaired, two-tailed *t*-test (^*∗*^ *P* < 0.05).

Three goslings were randomly sampled on day 1, day 3, and day 7 after infection with H9N2. QRT-PCR analysis found that goose TRIM25 expression levels were significantly downregulated in H9N2-infected gosling trachea. On day 1 after infection, the transcription levels were downregulated in the blood and the transcription levels were downregulated in blood and brain on the day 3 after infection. The mRNA levels of goose TRIM25 in the lungs were significantly upregulated on day 7 after infection (Figure 7).

## 4. Discussion

The innate immune signaling pathways, such as RIG-I, are a critical component of the antiviral response. TRIM25 has a significant impact on the RIG-I signaling pathway. First, after RIG-I is activated following PAMP RNA recognition, TRIM25 modifies RIG-I with ubiquitin. Then, CARD domains of RIG-I and MAVs conjugate to form a tetramer. Finally, the interactions between the CARD domains of RIG-I and MAVs activate TBK1 and IKK*ε* to initiate downstream signaling [24–27]. It was reported that TRIM25 exists in fish, humans, mice, chickens, and ducks. Domingo Miranzo-Navarro indicated that duck TRIM25 is an important component of the antiviral response by demonstrating that TRIM25 modifies RIG-I through ubiquitination, which was reported recently not to be related to ubiquitin anchoring [10]. Aquatic birds, particularly geese, contribute significantly to the transmission of many major pathogens, including AIV [28]. Though the complete genome of geese has been sequenced, many antiviral genes of geese are still unknown, including TRIM25.

As with many other TRIM family proteins, TRIM25 has a conserved N-terminal region RBCC motif that consists of a RING finger domain, one or two B-box domains and a coiled-coil domain [3, 29]. The RING finger domain is defined by the following consensus sequence CX2CX(9–39)CX(1–3)HX(2-3)C/HX2CX(4–48)CX2C. The Cys and His residues form a cloud bond with the two zinc atoms and function as an E3-ubiquitin ligase (an enzyme involved in ubiquitination) [30]. This interaction between the E3-ubiquitin ligase and its target proteins is required for the antiviral response of most functional TRIM proteins. The B-box is also a zinc-binding motif, which is found exclusively in TRIM proteins [31, 32] and reportedly also functions as an E3 ligase [33]. The coiled-coil domain, the last domain of the RBCC, mediates the homodimerization/oligomerization in a large number of TRIM proteins, which may contribute to the diversity of their biological functions [34–36]. Mutation and deletion experiments have revealed that the coiled-coil domain may also be important for the formation of subcellular structures, including cytoplasmic or nuclear bodies (NBs) [35]. In general, the RING domain functions as an E3 ligase, while the C-terminal PRY/SPRY domain determines the specific target proteins for TRIM ubiquitination [2].

The 1892 bp full length goose TRIM25 cDNA cloned and sequenced in this study contained a 1662 bp ORF. It is longer than the predicted TRIM25 cDNA of* Anser cygnoides* listed in the GenBank database (accession numbers XM_013178947, XM_013178948). The predicted* Anser cygnoides* TRIM25 transcript variant X1 had a 1557 bp (transcript variant X2: 1638-bp) ORF with a short 5′UTR and a long 3′UTR, encoding 519-amino acid (transcript variant X2: 545-amino acid). The goose TRIM25 was cloned from gosling lung tissue, which had an RBCC motif in the N-terminal domain and a PRY/SPRY in C-terminal domain. Interestingly, the RING finger domain present in* Anas platyrhynchos* (XM_013092756.1) and* G. gallus* (XM_415653.4) is the most critical domain for E3-ubiquitin ligase activity [30]. However, according to our data (repeat 5RACE-PCR many times), the RING finger domain was absent in goose TRIM25 cDNA, which was consistent with the predicted goose TRIM25 sequence. Although the TRIM25 RING finger domain was suggested by previous studies to play an important role in the innate antiviral immune response [11, 20], one study suggested that B-Boxes could provide an E3 binding site, similar to the RING domain, thereby conferring E3 ligase activity to RINGless TRIMs [23]. It was revealed that the same E3 ligase activity was detected in the RING finger domain-deleted TRIM16 in vitro [37]. These data suggest that goose TRIM25, although lacking a RING domain, had the capacity to act as an E3 ligase and may function as an antiviral gene; however, this remains to be determined.

One could conclude from the tissue distribution profiles of goose TRIM25 that immune-associated tissues, such as blood, bursa of Fabricius, and harderian gland, had relatively high TRIM25 expression levels. In addition, it was more highly expressed in the tissues obtained from goslings when compared to those from adult geese. We found that TRIM25 expression levels were the highest in the blood of both geese and goslings; however, high TRIM25 transcription levels were also observed in gosling proventriculus and adult goose liver. It differed from the expression pattern of* G. gallus* TRIM25 where the highest levels were found in the lungs [20]. In contrast to the results of the current study, a previous study found that the highest TRIM transcription levels in felines were found in the heart [18].

ODN2006 is a synthesized oligonucleotide, determined in our previous study, is recognized by goose TLR21, and subsequently upregulates the expression of IFN [38]. Poly(I:C) is a synthesized oligonucleotide that not only functions as a TLR3 ligand but also activates the RIG-I and MDA5 signaling pathways, inducing the expression of IFN*α* [39, 40]. R848 is a TLR agonist, which previously has been used as a vaccine adjuvant. We found that there was significant upregulation of TRIM25 expression in ODN2006, poly(I:C), R848, and PBMCs treated with IFN*α* for 6 hours. It was reported that the TRIM family proteins could be induced by IFNs in humans [41]. This study investigated if goose IFNs, including IFN*α*, IFN*γ,* and IFN*λ* which were determined to be expressed in transfected BHK 21 in our previous study, can trigger goose TRIM25 expression. As expected, the expression of goose TRIM25 was significantly upregulated 24 hours after treatment with goose IFN*α*, IFN*γ*, and IFN*λ* protein in GEFs, which is consistent with findings reported in mammals.

Anna Figuiredo recently reported that KAP1, one of the TRIM family proteins, inhibits HIV-1 integration [14]. An increasing number of TRIM proteins were determined to contribute to the antiviral response by mediating the antiviral signaling pathways in mammals [12, 42]. TRIM family proteins play a critical role in the innate immune antiviral response. Duck TRIM25 was found to play an important role in RIG-I-dependent antiviral signaling pathways [9]. QRT-PCR revealed that gosling TRIM25 expression levels were significantly downregulated in the trachea of H9N2-infected birds 1 day after infection and in the blood, brain, and small intestines 3 days after infection. As reported, the low pathogenic avian influenza virus is sensed by host TLRs, which in turn reduce the expression of IFNs to defend the host against the infection [43, 44]. Our in vitro study showed that the goose TRIM25 transcription levels were significantly upregulated by H9N2 and GPV treatment, which is consistent with previous studies [14, 19, 20, 45].

Interestingly, the differential expression of goose TRIM25 was detected in the H9N2-infected geese and PBMCs, which coincides with our previous findings. We designed an experiment in which the transcription levels of IFN*α* and IFN*γ* were measured in goslings and PBMCs infected with H9N2. The results revealed that IFN*α* expression was inhibited in the immune-related tissues of H9N2-infected goslings, such as the harderian gland and the bursa of Fabricius, while in PBMCs it was upregulated [45]. And the goMx transcription levels were upregulated in the trachea at 3 d.p.i. but downregulated in small intestine [46]. We speculate H9N2 utilized some unknown mechanism to evade the host immune response and decrease the production of IFN*α* and eventually decrease the production of TRIM25. Some reports indicated that the NS of the low pathogenic avian influenza virus can inhibit the host antiviral response and reduce IFNs production [47, 48]. These reports are consistent with our speculation.

## 5. Conclusion

In this study, we cloned goose TRIM25, determined its tissue distribution profiles, described its nucleotide sequence, and conducted both structural and phylogenetic analyses. The cloned full length goose TRIM25 cDNA contained a 1662-bp ORF but lacked the E3 ligase RING domain. QRT-PCR results revealed that goose TRIM25 is highly expressed in immune-associated tissues. TRIM25 transcription was induced by goose IFNs, poly(I:C), and ODN 2006, R848, GPV in H9N2-treated GEFs or PBMCs; however, it was inhibited in H9N2-treated goslings. A tentative conclusion could be drawn that goose TRIM25 transcription levels could be induced by goose IFN*α*, goose IFN*γ*, and goose IFN*λ*, as well as poly(I:C), ODN 2006 and R848 in vitro, but it is inhibited in H9N2-infected gosling through some unknown mechanism.

## Figures and Tables

**Table 1:** The list of primers.

Name	Primer sequence 5′-3′
G-TRIM25-F	GGTGCAGACGTGCCTGAC
G-TRIM25-R	CAGACCAAAGAATCATCAG
G-TRIM25-RT-F	CCACCACCCTCAGCGTTTC
G-TRIM25-RT-R	GCCATAGCAGATGCCAAT
*β*-Actin-F	CCGTGACATCAAGGAGAA
*β*-Actin-R	GAAGGATGGCTGGAAGAG

## Abbreviations

RIG-I: Retinoic acid-inducible gene I
RLRs: Retinoic acid-inducible gene I- (RIG-I-) like receptors
IFNs: Interferon
TRIM25: The tripartite motif containing 25 proteins
CARDs: Caspase activation and recruitment domains
MAVs: Mitochondria antiviral signal molecules
ORF: Open reading flame
Poly(I:C): Polyinosinic-polycytidylic acid
ODN: Oligodeoxynucleotides
R848: Resiquimod
PRRs: Pattern recognition receptors
TLRs: Toll-like receptors
NLRs: NOD-like receptors
PAMP: Pathogen-associated molecular pattern
NEMO: NF-*κ*B essential modulator
TRAF3: Tumor necrosis factor receptor associated factor 3
IRF3: Interferon regulatory factor 3
qRT-PCR: Quantitative reverse transcription polymerase chain reaction
ISG 15: Interferon stimulated gene 15
PBMCs: Peripheral blood mononuclear cell
GEFs: Goose embryo fibroblasts
UTRs: Untranslated regions
DMEM: Dulbecco's modified Eagles medium
BHK 21: Baby hamster kidney 21 cells
ATP: Adenosine triphosphate
GPV: Gosling plague virus.

## References

[1] Nisole S., Stoye J. P., Saïb A. (2005). TRIM family proteins: retroviral restriction and antiviral defence. *Nature Reviews Microbiology*.

[2] Rajsbaum R., García-Sastre A., Versteeg G. A. (2014). TRIMmunity: the roles of the TRIM E3-ubiquitin ligase family in innate antiviral immunity. *Journal of Molecular Biology*.

[3] Meroni G., Diez-Roux G. (2005). TRIM/RBCC, a novel class of ‘single protein RING finger’ E3 ubiquitin ligases. *BioEssays*.

[4] Loo Y.-M., Gale M. (2011). Immune signaling by RIG-I-like receptors. *Immunity*.

[5] Wang X.-W., Wang J.-X. (2013). Pattern recognition receptors acting in innate immune system of shrimp against pathogen infections. *Fish & Shellfish Immunology*.

[6] Komander D., Rape M. (2012). The ubiquitin code. *Annual Review of Biochemistry*.

[7] Chen J., Chen Z. J. (2013). Regulation of NF-*κ*B by ubiquitination. *Current Opinion in Immunology*.

[8] Zeng W., Sun L., Jiang X. (2010). Reconstitution of the RIG-I pathway reveals a signaling role of unanchored polyubiquitin chains in innate immunity. *Cell*.

[9] Inn K.-S., Gack M. U., Tokunaga F. (2011). Linear ubiquitin assembly complex negatively regulates RIG-I- and TRIM25-mediated type I interferon induction. *Molecular Cell*.

[10] Miranzo-Navarro D., Magor K. E. (2014). Activation of duck RIG-I by TRIM25 is independent of anchored ubiquitin. *PLoS ONE*.

[11] Zhang F., Hatziioannou T., Perez-Caballero D., Derse D., Bieniasz P. D. (2006). Antiretroviral potential of human tripartite motif-5 and related proteins. *Virology*.

[12] Kawai T., Akira S. (2011). Regulation of innate immune signalling pathways by the tripartite motif (TRIM) family proteins. *EMBO Molecular Medicine*.

[13] Uchil P. D., Hinz A., Siegel S. (2013). TRIM protein-mediated regulation of inflammatory and innate immune signaling and its association with antiretroviral activity. *Journal of Virology*.

[14] Allouch A., Di Primio C., Alpi E. (2011). The TRIM family protein KAP1 inhibits HIV-1 integration. *Cell Host and Microbe*.

[15] Arimoto K.-I., Funami K., Saeki Y. (2010). Polyubiquitin conjugation to NEMO by triparite motif protein 23 (TRIM23) is critical in antiviral defense. *Proceedings of the National Academy of Sciences of the United States of America*.

[16] Stremlau M., Owens C. M., Perron M. J., Kiessling M., Autissier P., Sodroski J. (2004). The cytoplasmic body component TRIM5*α* restricts HIV-1 infection in Old World monkeys. *Nature*.

[17] Rajsbaum R., Stoye J. P., O'Garra A. (2008). Type I interferon-dependent and -independent expression of tripartite motif proteins in immune cells. *European Journal of Immunology*.

[18] Koba R., Kokaji C., Fujisaki G., Oguma K., Sentsui H. (2013). Characterization of feline TRIM genes: molecular cloning, expression in tissues, and response to type I interferon. *Veterinary Immunology and Immunopathology*.

[19] Miranzo-Navarro D., Magor K. E. (2014). Activation of duck RIG-I by TRIM25 is independent of anchored ubiquitin. *PLoS ONE*.

[20] Feng Z.-Q., Cheng Y., Yang H.-L., Zhu Q., Yu D., Liu Y.-P. (2015). Molecular characterization, tissue distribution and expression analysis of *TRIM25* in *Gallus gallus domesticus*. *Gene*.

[21] Magor K. E., Miranzo Navarro D., Barber M. R. W. (2013). Defense genes missing from the flight division. *Developmental and Comparative Immunology*.

[22] Koba R., Oguma K., Sentsui H. (2015). Overexpression of feline tripartite motif-containing 25 interferes with the late stage of feline leukemia virus replication. *Virus Research*.

[23] Versteeg G. A., Benke S., García-Sastre A., Rajsbaum R. (2014). InTRIMsic immunity: positive and negative regulation of immune signaling by tripartite motif proteins. *Cytokine and Growth Factor Reviews*.

[24] Kowalinski E., Lunardi T., McCarthy A. A. (2011). Structural basis for the activation of innate immune pattern-recognition receptor RIG-I by viral RNA. *Cell*.

[25] Jiang F., Ramanathan A., Miller M. T. (2011). Structural basis of RNA recogination and activation by innate immune receptor RIG-I. *Nature*.

[26] Gack M. U., Shin Y. C., Joo C.-H. (2007). TRIM25 RING-finger E3 ubiquitin ligase is essential for RIG-I-mediated antiviral activity. *Nature*.

[27] Hou F., Sun L., Zheng H., Skaug B., Jiang Q. X., Chen Z. J. (2011). MAVs forms functional prion-like aggregates to activate and propagate antivial innate immune response. *Cell*.

[28] Roach J. M., Racioppi L., Jones C. D., Masci A. M. (2013). Phylogeny of Toll-like receptor signaling: adapting the innate response. *PLoS ONE*.

[29] Short K. M., Cox T. C. (2006). Subclassification of the RBCC/TRIM superfamily reveals a novel motif necessary for microtubule binding. *The Journal of Biological Chemistry*.

[30] Joazeiro C. A., Weissman A. M. (2000). RING finger proteins: mediators of ubiquitin ligase activity. *Cell*.

[31] Meronin G., Diez-Roux G. (2005). TRIM/RBCC, a novel class of ‘single protein RING Finger’ E3 ubiquitin ligases. *BioEssays*.

[32] Pertel T., Hausmann S., Morger D. (2011). TRIM5 is an innate immune sensor for the retrovirus capsid lattice. *Nature*.

[33] Massiah M. A., Simmons B. N., Short K. M., Cox T. C. (2006). Solution structure of the RBCC/TRIM B-box domain of human MID1: B-box with a RING. *Journal of Molecular Biology*.

[34] Sardiello M., Cairo S., Fontanella B., Ballabio A., Meroni G. I. (2008). Genomic analysis of the TRIM family reveals two groups of genes with distinct evolutionary properties. *BMC Evolutionary Biology*.

[35] Reymond A., Meroni G., Fantozzi A. (2001). The tripartite motif family identifies cell compartments. *EMBO Journal*.

[36] Napolitano L. M., Meroni G. (2012). TRIM family: pleiotropy and diversification through homomultimer and heteromultimer formation. *IUBMB Life*.

[37] Bell J. L., Malyukova A., Holien J. K. (2012). TRIM16 acts as an E3 ubiquitin ligase and can heterodimerize with other TRIM family members. *PLoS ONE*.

[38] Qi Y., Yan B., Chen S. (2016). CpG oligodeoxynucleotide-specific goose TLR21 initiates an anti-viral immune response against NGVEV but not AIV strain H9N2 infection. *Immunobiology*.

[39] Kato H., Takeuchi O., Sato S. (2006). Differential roles of MDA5 and RIG-I helicases in the recognition of RNA viruses. *Nature*.

[40] Schwarz H., Schneider K., Ohnemus A. (2007). Chicken toll-like receptor 3 recognizes its cognate ligand when ectopically expressed in human cells. *Journal of Interferon and Cytokine Research*.

[41] Carthagena L., Bergamaschi A., Luna J. M. (2009). Human TRIM gene expression in response to interferons. *PLoS ONE*.

[42] Ozato K., Shin D.-M., Chang T.-H., Morse H. C. (2008). TRIM family proteins and their emerging roles in innate immunity. *Nature Reviews Immunology*.

[43] Xing Z., Cardona C. J., Li J., Dao N., Tran T., Andrada J. (2008). Modulation of the immune responses in chickens by low-pathogenicity avian influenza virus H9N2. *Journal of General Virology*.

[44] Ku K. B., Park E. H., Yum J. (2014). Transmissibility of novel H7N9 and H9N2 avian influenza viruses between chickens and ferrets. *Virology*.

[45] Zhou H., Chen S., Yan B. (2016). LPAIV H9N2 drives the differential expression of goose interferons and proinflammatory cytokines in both in vitro and in vivo studies. *Frontiers in Microbiology*.

[46] Zeng M., Chen S., Wang M. (2016). Molecular identification and comparative transcriptional analysis of myxovirus resistance GTPase (Mx) gene in goose (Anser cygnoide) after H9N2 AIV infection. *Comparative Immunology, Microbiology and Infectious Diseases*.

[47] Talon J., Horvath C. M., Polley R. (2000). Activation of interferon regulatory factor 3 is inhibited by the influenza a virus NS1 protein. *Journal of Virology*.

[48] Ludwig S., Wang X., Ehrhardt C. (2002). The influenza A virus NS1 protein inhibits activation of Jun N-terminal kinase and AP-1 transcription factors. *Journal of Virology*.

